# Closed-loop control of continuous piperacillin delivery: An *in silico* study

**DOI:** 10.3389/fbioe.2022.1015389

**Published:** 2022-10-20

**Authors:** Pau Herrero, Richard C. Wilson, Ryan Armiger, Jason A. Roberts, Alison Holmes, Pantelis Georgiou, Timothy M. Rawson

**Affiliations:** ^1^ Centre for Bio-Inspired Technology, Department of Electrical and Electronic Engineering, Imperial College London, London, United Kingdom; ^2^ Centre for Antimicrobial Optimisation, Imperial College London, London, United Kingdom; ^3^ National Institute for Health Research Health Protection Research Unit in Healthcare Associated Infections and Antimicrobial Resistance, Imperial College London, London, United Kingdom; ^4^ University of Queensland Centre for Clinical Research, Faculty of Medicine, The University of Queensland, Brisbane, QLD, Australia; ^5^ Herston Infectious Diseases Institute (HeIDI), Metro North Health, Brisbane, QLD, Australia; ^6^ The Departments of Pharmacy and Intensive Care Medicine, Royal Brisbane and Women’s Hospital, Brisbane, QLD, Australia; ^7^ Division of Anaesthesiology Critical Care Emergency and Pain Medicine, Nîmes University Hospital, University of Montpellier, Nîmes, France

**Keywords:** antimicrobials, beta-lactam, pharmacokinetics-pharmacodynamics, therapeutic drug monitoring, critical illness, closed-loop control

## Abstract

**Background and objective:** Sub-therapeutic dosing of piperacillin-tazobactam in critically-ill patients is associated with poor clinical outcomes and may promote the emergence of drug-resistant infections. In this paper, an *in silico* investigation of whether closed-loop control can improve pharmacokinetic-pharmacodynamic (PK-PD) target attainment is described.

**Method:** An *in silico* platform was developed using PK data from 20 critically-ill patients receiving piperacillin-tazobactam where serum and tissue interstitial fluid (ISF) PK were defined. Intra-day variability on renal clearance, ISF sensor error, and infusion constraints were taken into account. Proportional-integral-derivative (PID) control was selected for drug delivery modulation. Dose adjustment was made based on ISF sensor data with a 30-min sampling period, targeting a serum piperacillin concentration between 32 and 64 mg/L. A single tuning parameter set was employed across the virtual population. The PID controller was compared to standard therapy, including bolus and continuous infusion of piperacillin-tazobactam.

**Results:** Despite significant inter-subject and simulated intra-day PK variability and sensor error, PID demonstrated a significant improvement in target attainment compared to traditional bolus and continuous infusion approaches.

**Conclusion:** A PID controller driven by ISF drug concentration measurements has the potential to precisely deliver piperacillin-tazobactam in critically-ill patients undergoing treatment for sepsis.

## 1 Introduction

Precision antimicrobial dosing is an under-investigated area in the field of antimicrobial prescribing Rawson et al. (2021). Drug-resistance is increasing in many common infective organisms and is driving increased mortality Murray et al. (2022). *In vitro*, resistance to antimicrobials can be observed through determination of the minimum inhibitory concentrations (MIC). This describes the lowest concentration of antibiotic required to inhibit the visible growth of bacteria in standardised laboratory conditions Kahlmeter (2014). There is a clear relationship between worse treatment outcomes and elevated MIC when a standard antimicrobial dose is delivered Drusano (2004), Henderson et al. (2021). Optimising the dose of an antibiotic against the organisms MIC may therefore promote improved clinical outcomes and prevent the propagation of further drug-resistant mutants Drusano et al. (2015b), Drusano et al. (2015a), Vardakas et al. (2018).

Beta-lactam antimicrobials are the cornerstone of infection management. Piperacillin, a beta-lactam antimicrobial, is co-formulated with a beta-lactamase enzyme inhibitor, tazobactam, giving it a broad spectrum of activity against many hospital-acquired bacterial pathogens. Beta-lactams have a time dependent mechanism of action with the time the free, unbound drug concentration spends above the MIC (%*f*T > MIC) being the pharmacokinetic-pharmacodynamic (PK-PD) index associated with clinical outcome Abdul-Aziz et al. (2020). In critically ill patients, wide pharmacokinetic (PK) variability means that achieving adequate drug exposure using intermittent dosing can be challenging Roberts et al. (2014). Therefore, the use of prolonged or continuous infusions of piperacillin-tazobactam in critically ill patients has been recommended to improve %*f*T > MIC and maximise the clinical effectiveness Abdul-Aziz et al. (2020).

**FIGURE 1:**
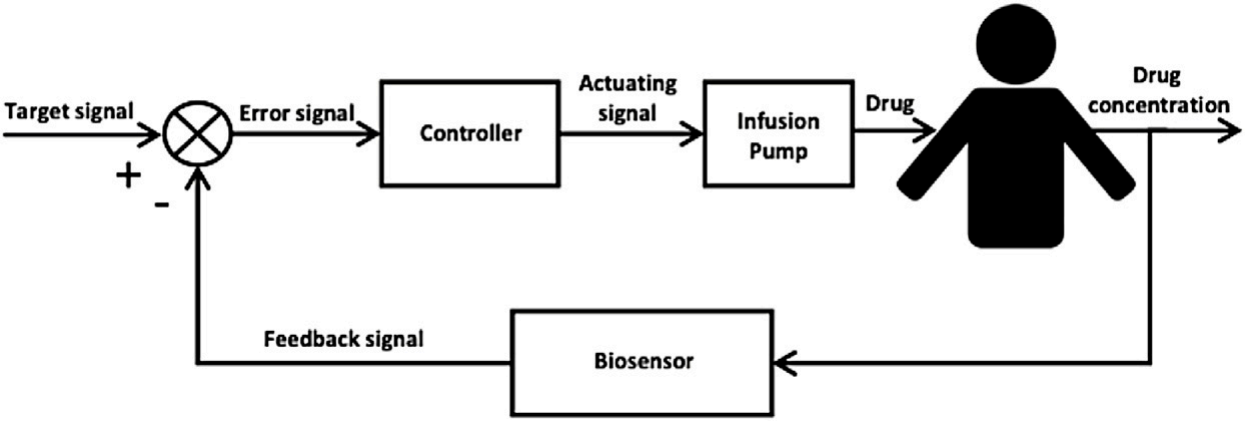
Block diagram of a closed-loop control system for automated, precision drug delivery incorporating a biosensor, a feedback controller and an infusion pump for drug deliver to the patient. The biosensor provides real-time *in vivo* measurements of the patient’s drug concentration level which is then compared to a predefined target signal (set-point) to produce an error signal that is fed to the feedback controller. The feedback controller by its design computes a corrective action to reduce the error signal and sends an actuating signal to the infusion pump which delivers the corresponding drug dose to the patient.

In patients with sepsis, continuous or prolonged infusions of beta-lactams have been associated with improved clinical outcomes Vardakas et al. (2018). However, the precision with which gold standard PK-PD targets are achieved by infusion is not routinely assessed. Even applying therapeutic drug monitoring to guide continuous infusion dosing, leads to a significant number of patients not reaching desirable concentration targets Hagel et al. (2022). Research into the role of closed-loop control for the delivery of optimal antimicrobial dosing has been previously explored for vancomycin, a glycopeptide antibiotic with a narrow therapeutic window Herrero et al. (2017). Closed-loop control has been demonstrated as an effective intervention in several fields of medicine including glucose control using the artificial pancreas system and anaesthesia delivery. Bekiari et al. (2018), Ghita et al. (2020) Closed-loop control for drug delivery uses feedback, typically from an electrochemical sensor, to adjust the delivery of a drug to minimise error in target attainment (Figure 1) Yu et al. (2018). For vancomycin, a proportional-integral-derivative (PID) controller was able to achieve significantly greater time within target range compared to standard therapeutic drug monitoring (TDM) approaches *in silico* Herrero et al. (2017).

Given the importance of optimising beta-lactam antibiotic delivery, we developed a closed-loop control system for the precision delivery of piperacillin with tazobactam by continuous infusion in critically ill patients.

## 2 Materials and methods

### 2.1 Piperacillin pharmacokinetic model

Data from 20 septic critically ill patients from a previously published prospective PK study with piperacillin-tazobactam were used in this study Udy et al. (2015). Only patients with both microdialysis and serum monitoring were included. All patients received 4.5 g intravenous (IV) infusions of piperacillin-tazobactam over 20 min at 6-h intervals. Patients underwent blood sampling during a single dosing interval at 1 min pre-dose, 20, 40, 60, 210 and 360 min after the observed dose. Patients underwent subcutaneous tissue microdialysis (CMA 60, 20 kDa dialysis window, Global Scientific, Sweden). Microdialysis samples were taken 1 min pre-dose with repeat measurements taken at 20, 40, 60, 90, 120, 150, 180, 210, 240, 270, 300, 330, and at 360 min. Samples were spun and stored at −80°C prior to analysis. Total and unbound piperacillin concentrations were determined using ultrafiltration (Centrifree 30,000 NMWL; Merck Millipore, Tullagreen, Ireland) following a standardised methodology Udy et al. (2015). Piperacillin concentration was determined using a validated HPLC-MS/MS methodology with a lower limit of quantification of 1 mg/L. Concentrations of tazobactam were not measured. Patients were excluded if they had renal impairment (plasma creatinine concentration greater than 171 *μ*mol/L) or needed renal replacement therapy. Mean creatinine clearance was 98.4 ml/min with a standard deviation of 50.2.

**FIGURE 2:**
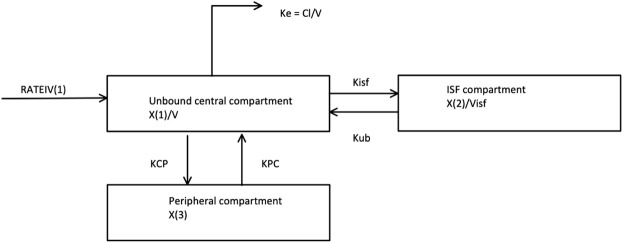
The Piperacillin pharmacokinetic compartment model selected and fit to simulate the 20 virtual critically-ill patients. This is a 3 compartment model with an unbound central, ISF and peripheral compartment. RATEIV is the rate of the IV drug delivery. KCP and KPC are transfer coefficients to and from the Peripheral compartment, respectively. Kisf and Kub are transfer coefficients to and from the ISF compartment, respectively. V is the volume of the central compartment. Visf is the volume of the ISF compartment. X (1), X (2) and X (3) are the amount of the drug in the Unbound, ISF and Peripheral compartments respectively. Ke is the elimination rate constant.

For PK model development, 2-, 3-, and 4-compartment models were fitted to the data using Pmetrics within Neely et al. (2012) Total piperacillin concentration was omitted from the modelling process. The fit of models to data were evaluated using 1) the coefficients of determination (*R* ^2^), the y-intercept, and slope of regression from observed—versus predicted plots before and after the Bayesian plot; 2) the log-likelihood value; 3) Akaike information criteria (AIC); and 4) Normalised Prediction Distribution Error (NPDE) analysis. Given that only a small number of patients were included in this analysis, co-variate modelling was not performed. The final model is described in Figure 2 (full parameter estimates can be found in Supplementary Material S3).

### 2.2 Closed-loop controller

Closed-loop control systems work on the principle of feedback in which a signal (e.g., drug concentration) to be controlled is compared to a reference signal (e.g., therapeutic drug concentration). The error between the two signals is then used to tabulate a corrective action (e.g., increase, maintain, or decrease drug dosage). Figure 2 shows a block diagram of a generic closed-loop control system for drug delivery. In this work, the subcutaneous route (i.e., interstitial fluid) is assumed to be accessible for measuring the antimicrobial levels, and the intravenous route is used for delivering the antibiotic.

Proportional-integral-derivative (PID) control was chosen for its simplicity and previous success in different drug delivery applications Steil (2013), Herrero et al. (2017), van Heusden et al. (2019). Furthermore, the large inter-individual PK variation observed and a paucity of covariate data with which to individualise our model makes it challenging for model-based control approaches, such as Model Predictive Control Krieger and Pistikopoulos (2014).

The employed PID control strategy is described by (Eq. 1).

$$u\left(t\right)=B\left(t\right)+K_{p}e\left(t\right)+K_{i}\int_{t=t-w}^{t}e\left(\tau \right)d\tau +K_{d}\frac{d_{e}\left(t\right)}{dt},$$ (1)

where *u*(*t*) represents the rate of infusion into the central compartment (e.g., blood); *B*(*t*) is a predefined infusion profile rate; *e*(*t*) is the error between the measured control variable (i.e., drug concentration) (*C*_*V*_(*t*)) and the setpoint (*SP*(*t*)) (i.e., *e*(*t*) = *C*_*V*_(*t*) − *S*_*P*_(*t*)); *K*_*p*_, *K*_*i*_ and *K*_*d*_ denote the coefficients for the proportional, integral, and derivative terms, respectively; and w is a predefined time window length. Finally, the coefficients of the PID controller are defined as *K*_*d*_ = *K*_*p*_/*T*_*d*_ and *K*_*i*_ = *K*_*p*_ *T*_*i*_, where *T*_*d*_, and *T*_*i*_ are tunable time constant parameters.

The PID controller was discretized using a sampling period (30 min) proven to be adequate to control the antimicrobial PK observed in critically ill patients with rapidly changing physiology. In particular, the derivative of the error was approximated using backward differencing, and the integral term was approximated using the Euler’s method Biswas et al. (2013).

The discretised version of the employed PID controller is described by (Eq. 2).

$$u\left(k\right)=B\left(k\right)+K_{p}e\left(k\right)+K_{i}\overset{k}{\underset{k-w/\Delta t}{\sum }}e\left(k\right)+K_{d}\frac{e\left(k\right)-e\left(k-1\right)}{\Delta t},$$ (2)

where *k* represents the time instant and Δ*t* is the sample time (e.g., 30 min).

To minimise the chances of over- and under-delivery, a set of safety constraints were applied. In particular, u(k) was saturated if above 5 times the basal rate, or if below 50% of the previous rate of infusion; the delivery rate was halted if concentration was above the upper bound of the predefined therapeutic target range; to constrain the integral from reaching excessively high values, a sliding window of *w* = 6 h was employed; and the integral term was reset to zero whenever the sensor error was greater than 30%.

Finally, the controller’s set point was defined as the midpoint of a predefined target range [(20, 100) mg/L]. This range was selected to represent the worst-case scenario for empiric antimicrobial dosing, considering that the epidemiological cut-off for *Pseudomonas aeruginosa* is 16 mg/L. This range would therefore allow for dosing at 100% *f*T > MIC, an accepted minimum target for critically ill patients. Furthermore, it would facilitate more aggressive dosing (e.g., 100% *f*T > 4xMIC) whilst avoiding potential toxicity range, which has previously been reported at approximately 150 mg/L Abdul-Aziz et al. (2020), Quinton et al. (2017).

### 2.3 Sensor calibration

Due to the significant mismatch between the observed interstitial fluid (ISF) concentration and serum concentration, and since the goal is to control serum concentration while measuring at ISF compartment, a calibration strategy was required. In particular, the one-point calibration strategy described by (Eq. 3) was employed.

$$CV\left(k\right)=m\left(i\right)\cdot M_{\mathrm{isf}}\left(k\right),\;\;with\;m\left(i\right)=\frac{M_{\mathrm{serum}}\left(i-t\right)}{M_{\mathrm{isf}}\left(i-t\right)},$$ (3)

where *M*_*isf*_(*i*) and *M*_*serum*_(*i*) are the interstitial and serum measurements at calibration time instant *i*. *t* is the delay in serum measurement turnaround.

A delay of 1 h in the lab turnaround of serum measurements is assumed, based on in-house experience of beta-lactam assays when deployed as a dedicated service as part of a closed-loop clinical trial. This is matched to the ISF measurement from the same time when calibrating. We assumed a 5% error on the serum concentration measurements used for calibration to account for instrumentation error. 5% error was chosen to coincide with FDA criteria for an acceptable chromatographic assay Food and Drug Administration (2018).

Due to the large intra-individual and inter-individual PK variation in critically ill patients and a paucity of co-variate data at the start of the treatment, an adaptive control strategy is proposed to individualise, in real time, the tuning of the controller (Supplementary Material S2).

### 2.4 *In silico* trials

To evaluate the performance of the proposed controller, simulation of 20 virtual critically-ill patients with highly variable antimicrobial PK was performed. For this purpose, the PK model described in Section 2.1 was employed. This is a common sample size for *in silico* trials of automated insulin delivery systems in diabetes management Sanz et al. (2021).

Individuals received a continuous infusion commencing with a loading dose of 4,000 mg piperacillin (with 500 mg tazobactam) followed by a modulated infusion of piperacillin-tazobactam by the controller for 30-h which started two and a half hours after the loading dose was delivered. Two target serum concentrations were explored based on achieving a 100%*f*T > MIC. Targets selected were a tight range of 32–64 mg/L (2-4x breakpoint of *P. aeruginosa*), which was used for evaluation purposes, and a broader range of 20–100 mg/L, which was used for control.

The initial concentrations in the central and peripheral compartments of the model used were assumed to be 0 mg/L. The duration of the simulation was 30-h and the sensor sampling time from ISF was assumed to be every 30 min. To introduce additional intra-day variability, renal clearance variability was artificially introduced by randomly generating a sinusoidal profile for each patient as described in Supplementary Material S4.

The initial piperacillin infusion rate was assumed to be 300 mg/h. Both the *in silico* environment including the PK model and the PID controller were implemented in Matlab 2021a (Mathworks). The PK model was simulated using the Matlab ode45 solver. The PID controller parameters were initialised to *K*_*p*_ = 5, *T*_*d*_ = 1, and *T*_*i*_ = 0.5. These values were manually tuned to achieve the overall best performance over the 20 virtual subjects.

**FIGURE 3:**
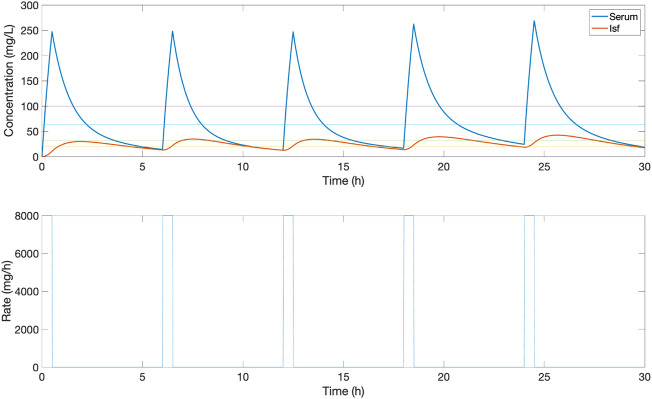
An example of the serum and ISF concentration (mg/L) of piperacillin and corresponding IV rate (mg/h) for an *in silico* patient dosed by protocol 1, a standard clinical dosing protocol where boluses of 4,000 mg are delivered at 6-h intervals. In this patient, this method provides a low serum time in range, due to peaks and troughs outside the 32–64 mg/L target evaluation range.

**FIGURE 4:**
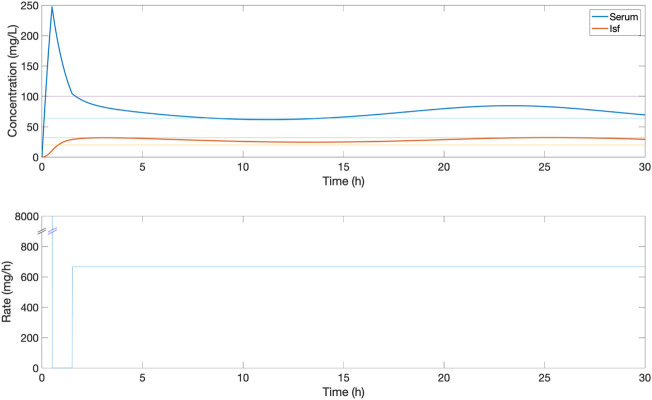
An example of the serum and ISF concentration (mg/L) of piperacillin and corresponding IV rate (mg/h) for an *in silico* patient dosed by protocol 2, a standard clinical dosing protocol where a loading dose of 4,000 mg is followed by a fixed continuous rate of 4,000 mg/6 h. In this patient, this continuous dose leads to serum concentrations above the 32–64 mg/L target evaluation range.

**FIGURE 5:**
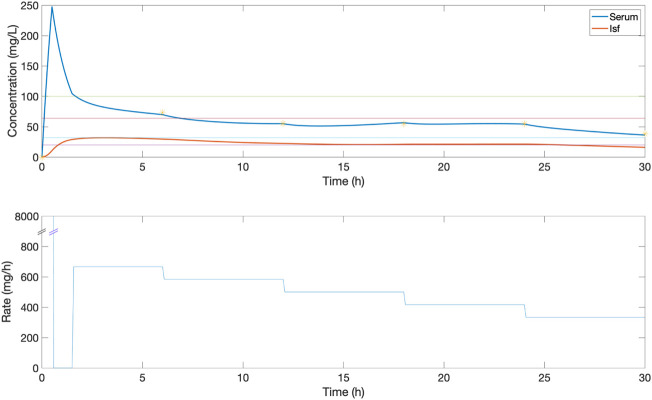
An example of the serum and ISF concentration (mg/L) of piperacillin and corresponding IV rate (mg/h) for an *in silico* patient dosed by comparison protocol 3, where a loading dose of 4000 mg is followed by a continuous rate of 4,000 mg/6 h which is titrated every 6 h by ± 500 mg if concentration was below or above the centre of target range 20–100 mg/L. Yellow stars show the value of measurements used to titrate, based on the 1 h delay. In this patient, dose titration improves serum time in the 32–64 mg/L evaluation range, with some variation within the range.

The proposed closed-loop controller was compared versus three standard clinical protocols for delivering piperacillin (with tazobactam). The first protocol (protocol 1) consisted in delivering 4,000 mg bolus at 6-h intervals, and the second protocol (protocol 2) consisted in delivering an initial loading dose of 4,000 mg and then delivering a fixed continuous rate of 4,000 mg/6-h. The third protocol (protocol 3) is the same as protocol 2 but titrating the continuous rate every 6-h based on drug level by ±500 mg if concentration was below or above the centre of target range 20–100 mg/L. For protocol 3, serum measurements from 1 h previously are used to titrate the dosage, to simulate delays in lab result turnaround. Example profiles for protocol 1, 2 and 3 can be found in Figures 3–5 respectively.

### 2.5 Statistical analysis

Pairwise Wilcoxon signed rank tests, as implemented in Matlab, were employed to compare the four interventions. The Holm-Bonferroni method was used to correct for the Family-wise Error Rate (*α* = 0.05).

## 3 Results

**FIGURE 6:**
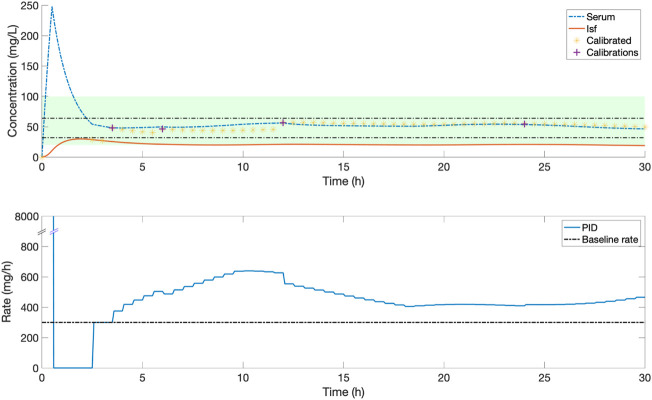
An example of the serum and ISF concentration (mg/L) of piperacillin and corresponding IV rate (mg/h) for an *in silico* patient dosed by closed-loop control. Yellow stars indicate the ISF sensor measurements after they have been calibrated to serum concentrations, purple crosses are the serum measurements used for this calibration. The shaded green zone is the target range, and black horizontal lines indicate the 32–64 mg/L evaluation range. In this patient, frequent adjustments by a closed-loop control system maintain a stable serum concentration within the evaluation range.

Figure 6 demonstrates an example of simulated closed-loop control for a selected individual patient receiving piperacillin-tazobactam. There is a significant mismatch between serum (blue line) and ISF (red line) concentration-time profiles. Population-PK modelling of piperacillin using a three-compartment model demonstrated wide inter-individual PK variability with mean (SD, % coefficient of variance) *Vd* 17.0 (8.0, 47%) L, *CL* 14.0 (8.4, 59.9%) L/h, and *Visf* 12.4 (12.8, 102.7%) L (Supplementary Material S3).

**FIGURE 7:**
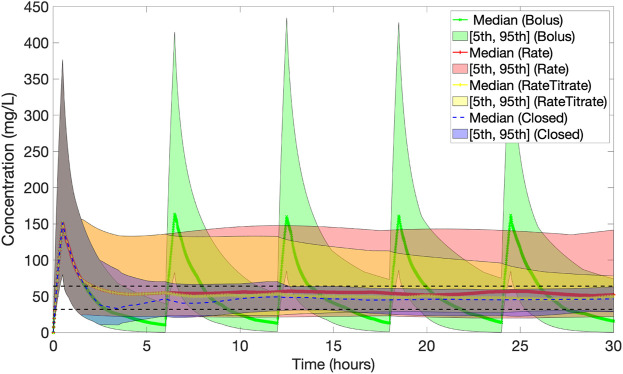
Population results [Median (5, 95)%] of piperacillin concentration (mg/L) over time corresponding to dosing by the PID controller (Closed) (in blue) together with the results for baseline protocols 1 (Bolus) (in green), 2 (Rate) (in red) and 3 (Rate Titrated) (in yellow). The black dashed lines indicate the 32–64 mg/L evaluation range. The PID controller reduces the population variability the most.

Figure 7 presents the population results [Median (5, 95)%] corresponding to the PID controller (Closed) together with the results for protocol 1 (Bolus), protocol 2 (Rate) and protocol 3 (Rate Titrate). Table 1 summarises the characteristics, therapeutic target attainment [(32, 64) mg/L], and median concentrations achieved using the closed-loop controller compared to protocol 1, 2 and 3.

**TABLE 1:** Comparison of protocols 1, 2, and 3 with closed-loop control for piperacillin-tazobactam delivery.

Intervention	MEAN	TIR32-64	TBR32	TAR64	TA32	DOSE
Protocol 1 bolus (B)	53.5^(*R*)^ [35.1, 68.0]	23.1^(*RT*,*C*)^ [19.5, 30.7]	39.7^(*R*,*RT*,*C*)^ [24.7, 63.4]	33.1^(*RT*,*C*)^ [17.49, 3.2]	60.3^(*R*,*RT*,*C*)^ [36.5, 75.2]	671.1^(*R*,*RT*)^ [671.1, 671.1]
Protocol 2 rate (R)	59.1^(*B*,*C*)^ [39.0, 74.8]	40.0^(*RT*,*C*)^ [21.4, 76.4]	0.3^(*B*)^ [0.17, 30.6]	23.2^(*RT*,*C*)^ [2.61, 67.3]	99.6^(*B*)^ [69.3, 99.8]	771.1^(*B*,*C*)^ [771.11, 771.11]
Protocol 3 rate titrate (RT)	53.0^(*C*)^ [43.8, 62.6]	79.0^(*B*,*R*)^ [50.4, 90.3]	0.5^(*B*)^ [0.28, 7.3]	9.1^(*B*,*R*,*C*)^ [2.63, 33.1]	99.4^(*B*)^ [92.6, 99.7]	738.8^(*B*,*C*)^ [638.8, 905.5]
Closed (C)	48.9^(*R*,*RT*)^ [38.0, 54.6]	91.2^(*B*,*R*)^ [60.8, 92.9]	0.5^(*B*)^ [0.28, 25.4]	4.9^(*B*,*R*,*RT*)^ [2.49, 7.0]	99.4^(*R*)^ [74.5, 99.7]	667.1^(*R*,*RT*)^ [596.4, 775.5]

All values represented as median [interquartile range]; MEAN, mean concentration for individual patients; TIR32-64, percentage time in range 32–64 mg/L; TBR32, percentage time below 32 mg/L; TAR64, percentage time above 64 mg/L; TA32, percentage time above 32 mg/L; DOSE, median dose/hour; ^*B*^ = *p* < 0.05 compared to protocol 1—bolus; ^*R*^ = *p* < 0.05 compared to protocol 2—rate; ^*RT*^ = *p* < 0.05 compared to protocol 3—rate titrated; ^*C*^ = *p* < 0.05 compared to closed-loop controller.

The median [IQR] of the mean concentration of piperacillin delivered was lower using closed-loop control (49.4 [38.6, 54.9] mg/L) compared to protocol 1 (53.5 [35.1, 68.0] mg/L, *p* = 0.072), protocol 2 (59.1 [39.0, 74.8] mg/L, *p* = 0.003), and protocol 3 (53.0 [43.8, 62.6] mg/L, *p* < 0.001). Median percentage time [IQR] within the defined target range of 32–64 mg/L was also greater using closed-loop control (91.8 [60.9, 93.21] %) compared to protocol 1 (23.1 [19.5, 30.7] %, *p* < 0.001), protocol 2 (40.0 [21.4, 76.4] %, *p* = 0.001), and protocol 3 (79.1 [50.4, 90.3] %, *p* = 0.142). Median dose per hour using closed-loop control (669.9 [569.9, 783.5] mg/h) was similar to protocol 1 (671.1 [671.1, 671.1] mg/h, *p* = 1) and lower than was delivered as part of protocol 2 (771.1 [771.1, 771.1] mg/h, *p* = 0.04) and protocol 3 (738.8 [638.8, 905.5] mg/h, *p* < 0.001).

## 4 Discussion

This study presents an *in silico* proof-of-concept for the application of PID control, facilitated by ISF drug monitoring to optimise the delivery of the beta-lactam antibiotic, piperacillin (co-formulated with tazobactam), in critically-ill patients. PID with a piperacillin ISF biosensor demonstrated higher time within serum target range compared to current drug delivery methods including intermittent bolus dosing (protocol 1), fixed rate continuous infusion (protocol 2), and serum therapeutic drug monitoring (TDM) guided continuous infusion (protocol 3).

The use of prolonged and continuous infusions in critical illness has been demonstrated to improve clinical outcomes, through the optimisation of antimicrobial PK-PD Vardakas et al. (2018), Kondo et al. (2020), Roberts et al. (2016). However, fixed rate continuous infusion may not lead to optimal drug exposure, due to high PK variability in critical illness. High PK-variability can be driven by factors such as augmented renal clearance, increased capillary leak, or the requirement for organ replacement therapy Rawson et al. (2021). A recent multi-centre, randomised controlled trial of continuous infusion of piperacillin-tazobactam with (intervention) and without (control) serum TDM guided dose adjustment in critically-ill patients demonstrated improved PK-PD target attainment and a trend towards improved clinical and microbiological outcomes using TDM Hagel et al. (2022). However, even with TDM-guided dose adjustment, a significant proportion of patients did not achieve optimal PK-PD targets, suggesting the additional methods of controlling the continuous infusion of piperacillin-tazobactam are required to effectively deliver individualised therapy.

Within this study, a similar observation to that made by Hagel and colleagues is observed Hagel et al. (2022). Whilst TDM-guided dose adjustment of continuous piperacillin-tazobactam infusion was associated with a higher percentage time within target range compared to fixed rate infusion (40% versus 79%), serum TDM guided infusion led to significantly more time in concentrations above the target range. This is associated with the risk of beta-lactam toxicity. Despite controlling drug delivery using a simulated sensor device measuring ISF piperacillin concentration Rawson et al. (2019) in the face of wide PK-variability, the PID controller reported in this study obtained a greater percentage time within serum target range (92%) compared to TDM guided dose adjustment.

Use of a simple PID controller might be sufficient to optimise piperacillin-tazobactam delivery. PID control has been successfully applied in other drug delivery applications, such as automated insulin delivery for glycemic control in diabetes Steil (2013). Future work will include the investigation of more advanced closed-loop control techniques, such as model predictive control (MPC) Pinsker et al. (2018), and may offer the advantage of being multivariate and able to intrinsically account for time delays and constraints.

The proposed adaptive control mechanism (Supplementary Material S2) was not triggered during the performed *in silico* trials, hence the tuning parameters remained constant along the simulations and among the evaluated virtual subjects. Nonetheless, when a narrower therapeutic target was selected, the adaptation mechanism was required.

This study demonstrating a proof-of-concept for ISF driven closed-loop control of piperacillin-tazobactam has several limitations that must be acknowledged. This study was developed using a data set of 20 critically ill patients from a single institution, so it may not be generalisable to other settings or across the whole spectrum of critically ill patients. Lab turnaround for serum measurements was modelled as 1 h, but this is currently not routinely available in clinical practice. Although variability in renal clearance and assay error have been accounted for within the *in silico* trials, not all potential variables have been accounted for that may be present in a real-world situation such as sensor artefacts and pump occlusions. Work is ongoing to define the observed sensor error for minimally invasive ISF sensors, such as those employing microneedle-based technology for real-time beta-lactam monitoring Rawson et al. (2019), Gowers et al. (2019), Rawson et al. (2017). *In silico* trials are not a replacement for clinical trials. However, they can be useful in speeding up and reducing the cost of developing automated approaches to drug delivery. Finally, this controller only takes into account the optimisation of piperacillin delivery. Piperacillin-tazobactam is a fixed dose combination of piperacillin, a beta-lactam antibiotic, with tazobactam, a beta-lactamase inhibitor. The optimal PK-PD target for tazobactam may differ from piperacillin, meaning that the efficacy of treatment will likely not be truly optimised until tazobactam’s PK-PD properties are also considered.

## 5 Conclusion

A PID controller using ISF piperacillin measurement was able to improve the serum time within target range compared to current approaches of intermittent and continuous dosing in critically ill patients. Future work will explore the clinical impact that implementation of closed-loop control antimicrobial dose optimisation can have on clinical outcomes.

## Data Availability

The data analysed in this study was obtained from Roberts et al. (2016). Datasets are available on request: The raw data supporting the conclusions of this article will be made available by the authors, without undue reservation.
