# Supplementary material for: Closed-loop control of continuous piperacillin delivery: An in silico study
Source: Front Bioeng Biotechnol. 2022 Oct 20;10:1015389. doi: 10.3389/fbioe.2022.1015389 (PMC9631830; doi:10.3389/fbioe.2022.1015389)
Supplement: Supplementary file 1 [file DataSheet1.PDF]

frontiers

Supplementary Material

1 WILCOXON SIGNED-RANK P VALUES

2 ADAPTIVE CONTROL MECHANISM

The adaptation mechanism is triggered at sensor calibration time (i) and adjusts the PID settings and the basal rate by multiplying them by an adaptive gain.

The adaptation mechanism uses a metric based on the percentage time the control variable (CV) is above or below target range (i.e. [20, 100] mg/L) over the last 6-hour window. The adaptation mechanism is defined by (S1)-(S8).

$$K(i) = \frac{20}{mean(CV(k-12:k))} \quad if\ TBR(i) > 50\%, \tag{S1}$$

$$K(i) = \frac{100}{mean(CV(k-12:k))} \quad if\ TAR(i) > 50\%, \tag{S2}$$

$$K_p(i) = K(i) \cdot K_p(i-1), \tag{S3}$$

$$K_d(i) = \frac{K_p(i)}{T_d}, \tag{S4}$$

$$K_i(i) = K_p(i) \cdot T_i, \tag{S5}$$

$$Basal(i) = K(i) \cdot Basal(i-1), \tag{S6}$$

$$TBR(i) = \frac{n(CV(k-12:k) < 20}{n(CV(k-12:k))} \cdot 100, \tag{S7}$$

$$TAR(i) = \frac{n(CV(k-12:k) < 100}{n(CV(k-12:k))} \cdot 100, \tag{S8}$$

where operator n(x) is the number of elements in array x.

3 PHARMACOKINETIC MODEL

Equations (S9) - (S11) describe a three-compartment pharmacokinetic model with central (x_1), interstitial fluid (x_2), and peripheral compartments (x_3).

$$\frac{dx_1}{dt} = R(t) - (Ke + Kisf + KCP) \cdot x_1 + Kub \cdot x_2 + KPC \cdot x_3 \tag{S9}$$

$$\frac{dx_2}{dt} = Kisf \cdot x_1 - Kub \cdot x_2 \tag{S10}$$

$$\frac{dx_3}{dt} = KCP \cdot x_1 - KPC \cdot x_3 \tag{S11}$$

where $R(t)$ in mg/h is a zero-order infusion of piperacillin(-tazobactam) into the central compartment (S9). Piperacillin(-tazobactam) has a volume, V, in litres and is cleared (L/h) from the central compartment by

Intervention	MEAN	TIR32-64	TBR32	TAR64	TA32	DOSE
Rate/Bolus	P<.001	.066	.001	.648	.001	P<.001
Rate/Closed	.003	.001	.546	P<.001	.546	.040
Rate/Rate Titrate	.127	.002	.546	.011	.546	1.000
Bolus/Closed	.072	P<.001	P<.001	P<.001	P<.001	1.000
Bolus/Rate Titrate	.985	P<.001	P<.001	.031	P<.001	.040
Closed/Rate Titrate	P<.001	.142	.164	.002	.164	P<.001

Table S1. Post-Correction P values for pairwise Wilcoxon signed-rank tests

	Mean	SD	CV%
Cl	14.0289	8.404177	59.90617
V	16.99523	7.989557	47.01059
Visf	12.41851	12.75296	102.6932
kub	1.154436	0.561056	48.60004
kisf	0.575631	0.685547	119.095
KCP	4.439132	6.461858	145.5658
KPC	13.18056	7.614225	57.76861

Table S2. Distributions of the estimated population pharmacokinetic parameters.CL (L/h), V (L), $Visf$ (L), kub (h^{-1}), $kisf(h^{-1})$, KCP (h^{-1}), KPC (h^{-1})

a secondary variable, $Ke = \frac{Cl}{V}$. The ISF compartment has an apparent volume, $Visf$, in litres. $Kisf$, Kub , KCP and KPC are first-order transfer coefficients connecting the compartments. Equation (S9) describes the rate of change of the amount of piperacillin, in mg, in the central compartment. Equation (S10) describes the rate of change of piperacillin in the interstitial fluid. Equation (S11) describes the rate of change in a peripheral compartment.

Table S2 presents the distributions of the estimated population pharmacokinetic parameters.

4 RENAL CLEARANCE INTRA-DAY VARIABILITY

Additional intra-day variability affecting renal clearance was introduced by means of the time varying function

$$Cl(t) = A \cdot Cl_0 sin \frac{2\pi}{1440} t + 2\pi\phi, \tag{S12}$$

where Cl_0 is the identified clearance parameter, A is the amplitude of the variation and ϕ a randomly generated number per individual between 0 and 1. In particular, A was set to 0.2 to model a 20% variability in clearance.

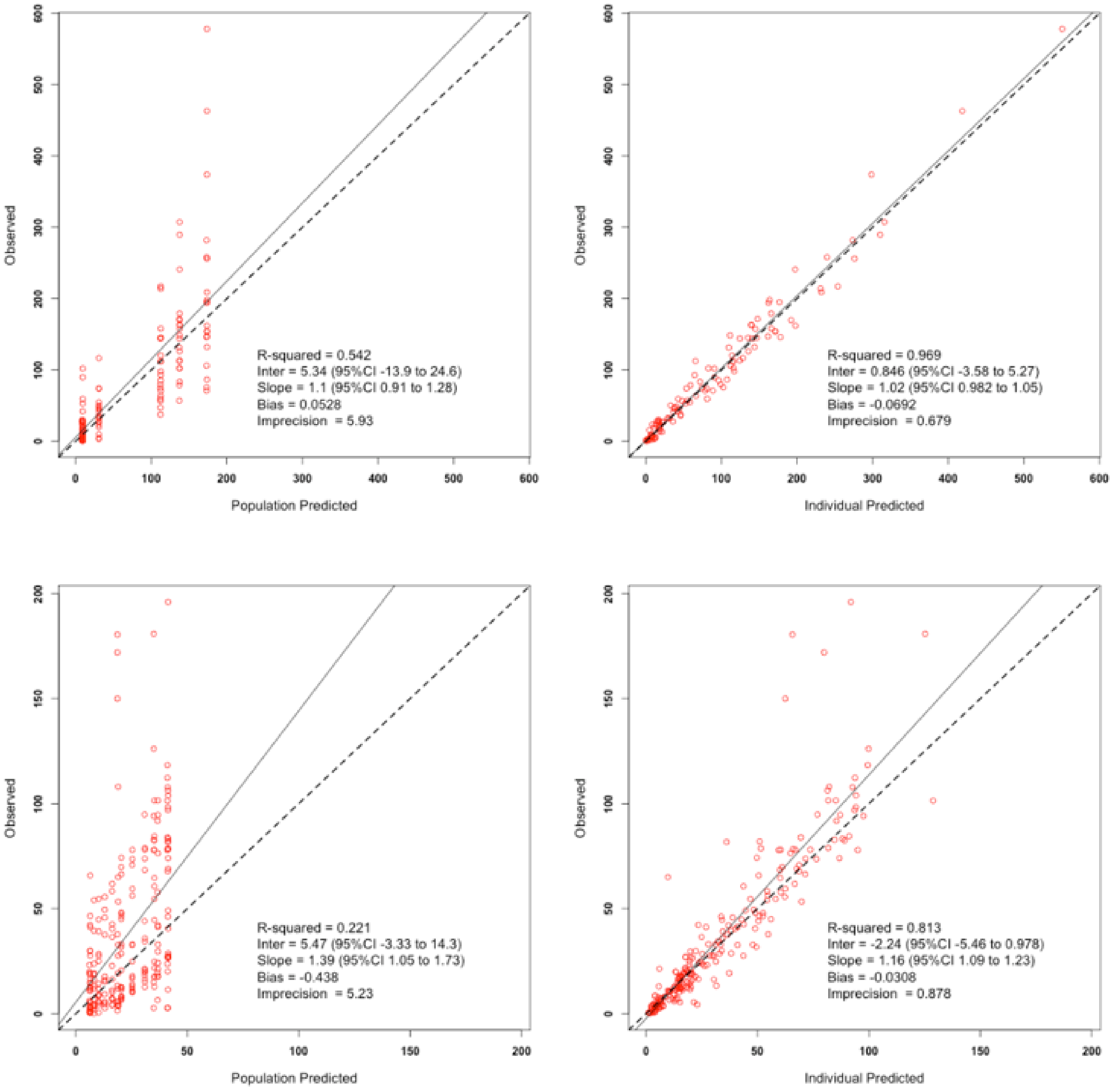

Figure S1. Individual and population posterior observed versus predicted plots for the piperacillin pharmacokinetic (PK) model
